# Focal monopolar pulsed field ablation for focal right atrial tachycardia: feasibility and safety from a single-centre experience

**DOI:** 10.1093/europace/euag129

**Published:** 2026-06-01

**Authors:** Ahmad Keelani, Obaida Alothman, Salome Tavkhelidze, Neha Krishnaswami, Georgi Borisov, Markus Frommhold, Hytham Abdelwahab, Santi Raffa, Dominik Linz, J Christoph Geller

**Affiliations:** Division of Cardiology, Arrhythmia Section, Zentralklinik, Robert-Koch-Allee 9, Bad Berka 99438, Germany; Division of Cardiology, Arrhythmia Section, Zentralklinik, Robert-Koch-Allee 9, Bad Berka 99438, Germany; Division of Cardiology, Arrhythmia Section, Zentralklinik, Robert-Koch-Allee 9, Bad Berka 99438, Germany; Division of Cardiology, Arrhythmia Section, Zentralklinik, Robert-Koch-Allee 9, Bad Berka 99438, Germany; Division of Cardiology, Arrhythmia Section, Zentralklinik, Robert-Koch-Allee 9, Bad Berka 99438, Germany; Division of Cardiology, Arrhythmia Section, Zentralklinik, Robert-Koch-Allee 9, Bad Berka 99438, Germany; Division of Cardiology, Arrhythmia Section, Zentralklinik, Robert-Koch-Allee 9, Bad Berka 99438, Germany; Division of Cardiology, Arrhythmia Section, Zentralklinik, Robert-Koch-Allee 9, Bad Berka 99438, Germany; Department of Cardiology, Cardiovascular Research Institute Maastricht (CARIM), Maastricht University Medical Centre, Maastricht, The Netherlands; Department of Biomedical Sciences, Faculty of Health and Medical Sciences, University of Copenhagen, Copenhagen, Denmark; Division of Cardiology, Arrhythmia Section, Zentralklinik, Robert-Koch-Allee 9, Bad Berka 99438, Germany; School of Medicine, Otto-von-Guericke University, Magdeburg, Germany

Catheter ablation is the most effective therapy in patients with atrial tachycardia.^1^ Right atrial tachycardia may be challenging because of proximity to the phrenic nerve and sinus node, limiting safety and efficacy. Pulsed field ablation (PFA) has improved the safety of atrial fibrillation (AF) ablation.^2,3^ Large-footprint PFA catheters have been used in right atrial tachycardia but may result in unnecessarily extensive lesions.^4^ Focal catheters may enable targeted lesion delivery while minimizing injury to the phrenic nerve or sinus node. However, current evidence is limited to case reports and heterogeneous series,^5–8^ and systematic data focusing on procedural workflow and safety of focal monopolar PFA in right atrial tachycardia—particularly in proximity to the phrenic nerve and sinus node—remain limited. This study evaluates the feasibility and safety characteristics of focal PFA for this indication.

In this retrospective study, we included consecutive patients who underwent catheter ablation for focal right atrial tachycardia using focal PFA at a single centre in Germany between November 2023 and November 2025. The study was approved by the local institutional review board.

Arrhythmias were induced using programmed atrial stimulation or isoproterenol infusion. Mapping was performed using three-dimensional (3-D) electro-anatomical mapping (EnSite NavX, Abbott, St. Paul, MN), a decapolar coronary sinus (CS) catheter, a contact-force ablation catheter, and either a duodecapolar Halo catheter or a high-density grid catheter. Focal PFA was delivered via an irrigated contact-force ablation catheter (TactiCath™, Abbott, St. Paul, MN) using the CENTAURI™ generator (CardioFocus, Marlborough, MA) that delivered monopolar, biphasic high-voltage pulse trains. The 25-Ampere (A) setting was used for standard energy delivery, consisting of 10 pulsed electric field trains with three packets per delivery. In regions demonstrating phrenic nerve capture, a reduced 22-A setting was applied, consisting of seven pulsed electric field trains with three packets per delivery. The sinus node region was delineated using 3-D electro-anatomical mapping when the tachycardia originated nearby, the phrenic nerve course was identified by high-output pacing.

A total of 15 consecutive patients were included (mean age 59 ± 12 years; 44% female). Structural heart disease was absent in all. Three patients had concomitant AF, and three had previously undergone radiofrequency ablation for right atrial tachycardia. During tachycardia, activation mapping identified the site of earliest atrial activation with a focal pattern, guiding targeted ablation at discrete right atrial sites. In 3/15 patients, left atrial mapping excluded a left-sided origin or confirmed pulmonary vein isolation (from previous ablation); in one patient, linear mitral isthmus ablation was performed using focal PFA with remapping and pacing manoeuvres confirming bidirectional block. Procedural endpoints included tachycardia termination and non-inducibility with isoproterenol infusion, followed by a 20–30 min waiting period.

Individual procedural characteristics, ablation settings, complications, and follow-up outcomes are summarized in *Table 1*. The most frequent sites of origin were the crista terminalis (*n* = 5), the inferolateral right atrium (*n* = 4), and the tricuspid valve ring (*n* = 3). In one patient each, the arrhythmia originated from the CS ostium, the posterior septal right atrium, and the SVC. Additional isolation of the SVC using a circumferential point-by-point approach with the focal PFA catheter was successfully performed in two patients due to frequent premature atrial contractions and paroxysmal AF.

**Table 1:** Procedural characteristics, ablation settings, complications, and follow-up outcomes in patients undergoing focal pulsed field ablation for focal right atrial tachycardia

Patient	Site of origin	PFA setting (Ampere)	PFA applications (n)	Additional ablation (if any)	Complication	Recovery/outcome	Follow-up (months)
1	Crista terminalis	25 A (22 A near phrenic nerve)	14	Isolation of the superior vena cava	Phrenic nerve palsy	Spontaneous recovery	No recurrence (Holter), 23 mo
2	Coronary sinus ostium	25 A (22 A near phrenic nerve)	21	Isolation of the superior vena cava	Sinus arrest and coronary vasospasm	Spontaneous recovery of sinus node function ST elevation with spontaneous recovery after intravenous nitroglycerine	No recurrence (Holter), 7 mo
3	Crista terminalis	25 A	8	—	—	—	No recurrence (Holter), 3 mo
4	Inferolateral right atrium	25 A	6	—	—	—	No recurrence (Holter), 21 mo.
5	Crista terminalis	25 A (22 A near phrenic nerve)	11	—	—	—	Asymptomatic (telephone follow-up), 25 mo.
6	Crista terminalis	25 A (22 A near phrenic nerve)	34	—	Sinus arrest	Spontaneous recovery of sinus node function	Recurrence of atrial tachycardia (Holter), 15 mo.
7	Inferolateral right atrium	25 A (22 A near phrenic nerve)	113	Anterior mitral isthmus line	—	—	No recurrence (Holter), 3 mo.
8	Inferolateral right atrium	25 A	9	—	—	—	No recurrence (Holter), 8 mo
9	Tricuspid valve ring	25 A	10	—	—	—	No recurrence (Holter), 3 mo.
10	Inferolateral right atrium	25 A	26	—	—	—	Recurrence of atrial tachycardia on device interrogation, 8 mo.
11	Crista terminalis	25 A (22 A near phrenic nerve)	8	—	—	—	No recurrence (Holter), 3 mo.
12	Tricuspid valve ring	25 A	8	—	—	—	Asymptomatic (telephone follow-up), 14 mo
13	Posterior septal right atrium	22 A	5	—	—	—	No recurrence (Holter), 7 mo.
14	Tricuspid valve ring	25A (22 A near phrenic nerve)	9	—	Phrenic nerve palsy	Spontaneous recovery	No recurrence (Holter), 7 mo
15	Superior vena cava	25 A (22 A near phrenic nerve)	17	—	—	—	No recurrence (Device interrogation), 6 mo

The mean procedure duration was 172 ± 65 min. The median number of PFA applications was 10 (IQR 8–21). Eleven procedures were performed without fluoroscopy; in the remaining cases, mean fluoroscopy time was 5 ± 5 min. Phrenic nerve capture during high-output pacing was observed in 8/15 patients (53%), either at the earliest activation site or during SVC isolation, prompting reduced-energy applications in these regions. Acute procedural success was achieved in all, with no acute recurrence during the waiting period, including sites treated with reduced-energy.

Periprocedural complications occurred in 4/15 patients. Transient sinus arrest occurred in two patients. In both cases, sinus node mapping had been performed, and ablation was delivered in proximity to the sinus node region. Sinus arrest with junctional escape rhythm lasted for a few minutes in both cases and resolved spontaneously during the procedure without the need for pacing or pharmacological support. Given the primarily non-thermal mechanism of PFA, this may reflect field spread with direct electrical effects on the sinus node, myocardial stunning, or autonomic modulation triggered by high-voltage pulse delivery. Transient phrenic nerve palsy occurred in one patient during additional SVC isolation at a site with phrenic capture and in one patient during focal ablation of the index tachycardia in proximity to the right phrenic nerve. Diaphragmatic function recovered before procedure completion, consistent with transient phrenic nerve stunning. In one patient, focal PFA at 25-A delivered without prophylactic nitroglycerine near the CS ostium resulted in acute coronary vasospasm with transient ST-segment changes, promptly resolving after intravenous nitroglycerine without haemodynamic compromise and hospitalization was two days.

Follow-up information was available for all patients (24-h Holter monitoring: *n* = 11, device interrogation: *n* = 2, and telephone follow-up: *n* = 2). During a median follow-up of 7 (IQR 3–14) months, tachycardia recurred in 2/15 patients (13%). The remaining patients had no arrhythmia recurrence, although asymptomatic episodes may have been missed. No late deaths or serious adverse events were observed.

Focal PFA has been increasingly applied in atrial arrhythmias. Prior reports have demonstrated feasibility in mixed atrial arrhythmias and complex atrial tachycardia populations. However, these studies predominantly involved left atrial and macro-re-entrant tachycardia and did not address focal right atrial tachycardia specifically.^7,8^

This early descriptive experience of focal PFA for focal right atrial tachycardia suggests feasibility. However, in this small cohort, the observed periprocedural complications are clinically relevant. All events were transient and resolved without permanent sequelae. However, their occurrence underscores the importance of careful application in anatomically sensitive regions. In this cohort, transient sinus arrest and coronary vasospasm occurred during focal ablation of the index tachycardia, whereas transient phrenic nerve palsy was observed during both focal ablation and more extensive lesion delivery for SVC isolation. On the other hand, despite phrenic capture in more than half of cases, focal PFA energy was delivered in regions where thermal ablation may be limited by concern for phrenic nerve injury. Future studies are warranted to further evaluate this approach.

## Data Availability

The data underlying this article will be shared on reasonable request to the corresponding author.
